# The Neutrophil/Lymphocyte Ratio Was Identified as a Marker of Severe Influenza During the 2024–2025 Outbreak in France

**DOI:** 10.3390/idr17050127

**Published:** 2025-10-10

**Authors:** Matteo Vassallo, Marion Derollez, Marc-Hadrien Veaute, Nicolas Clement, Roxane Fabre, Laurene Lotte, Yanis Kouchit, Sabrina Manni, Ursula Moracchini, Elea Blanchouin, Julie Better, Ludivine Rerolle, Raphael Chambon, Pierre Alfonsi Bertrand, Sarah Baccialone, Jerome Lemoine, Audrey Sindt, Pierre-Marie Bertrand

**Affiliations:** 1 Department of Internal Medicine Infectious Diseases, Cannes Hospital, 06400 Cannes, France; m.derollez@ch-cannes.fr (M.D.); veaute.mh@ch-nice.fr (M.-H.V.); s.manni@ch-cannes.fr (S.M.); u.warzochamoracchiniditmora@ch-cannes.fr (U.M.); e.blanchouin@ch-cannes.fr (E.B.); j.better@ch-cannes.fr (J.B.); l.rerolle@ch-cannes.fr (L.R.); 2 UR2CA (URRIS), Université Côte d’Azur, 06000 Nice, France; 3 Intensive Care Unit, Cannes Hospital, 06400 Cannes, France; n.clement@ch-cannes.fr (N.C.); y.khouchit@ch-cannes.fr (Y.K.); r.chambon@ch-cannes.fr (R.C.); p.alfonsibertrand@ch-cannes.fr (P.A.B.); s.baccialone@ch-cannes.fr (S.B.); j.lemoine@ch-cannes.fr (J.L.); pm.bertrand@ch-cannes.fr (P.-M.B.); 4 Public Health Department, UR2CA-RESPECT, 06000 Nice, France; fabre.r@chu-nice.fr; 5 Pain Management Unit and InovPain FHU, 06000 Nice, France; 6 Multipurpose Laboratory, Bacteriology and Virology Unit, Cannes Hospital, 06400 Cannes, France; la.lotte@ch-cannes.fr (L.L.); a.sindt@ch-cannes.fr (A.S.); 7 Aix Marseille Univ, CNRS, EFS, ADES, 13007 Marseille, France

**Keywords:** influenza, hospitalization, neutrophil/lymphocyte ratio, severity

## Abstract

Background/Objectives: Influenza continues to cause high morbidity and mortality rates worldwide, inflicting a major burden on the public health system. There is little data available on the 2024–2025 seasonal outbreak. Moreover, biomarkers for rapidly identifying subjects at higher risk for severe forms are needed. Methods: We retrospectively collected hospitalization data for influenza in Cannes, France, during the 2024–2025 seasonal outbreak. Severe forms were defined as cases either requiring admission to the Intensive Care Unit (ICU) or resulting in death. They were compared to uncomplicated forms. Main demographic, clinical, radiological, and laboratory characteristics were collected for each patient. Results: From October 2024 to May 2025, 59 patients were admitted to either the Infectious Diseases Department or the ICU (56% male, age 72 years, 27% vaccinated, influenza type A 93%, symptom duration 3.5 days prior to hospitalization, 31% admissions to ICU, 14% deaths). Vaccination status did not differ between severe and uncomplicated forms. In the univariate analysis, severe forms had higher neutrophil/lymphocyte and platelet/lymphocyte ratios upon admission and included more cases of acute hepatitis, pneumonia, and oseltamivir use than uncomplicated forms. A neutrophil/lymphocyte ratio > 15 was independently associated with severity (OR_adj_ 8.79, 95% CI: 1.34–57.6, *p* = 0.023), with 40.9% sensitivity, 94.6% specificity, 81.8% positive predictive value, and 72.3% negative predictive value for predicting a severe form. Conclusions: The N/L ratio was an easy-to-perform predictive marker for influenza severity during the 2024–2025 seasonal outbreak, warranting further prospective studies

## 1. Introduction

Influenza virus is the primary cause of acute respiratory tract infection worldwide, with annual epidemic outbreaks causing significant mortality and morbidity, especially among fragile populations [1,2,3,4], with a major impact on the public health system.

Elderly populations experience higher rates of severe forms, and it is estimated that influenza causes between 290,000 and 650,000 deaths worldwide each year [5].

Vaccines are currently the main preventive measure for managing seasonal influenza and reducing risks for severe forms [6]. However, vaccination rates are still insufficient [7], and their effectiveness varies according to seasonal clades [8].

Biomarkers for rapidly identifying subjects at higher risk for severe forms would be useful for clinicians, but data are limited. Indeed, interferon polymorphisms have been associated with different viral clearance capacities, but their use in clinical practice is complex [9]. In addition, the platelet distribution width and the neutrophil/lymphocyte and the platelet/lymphocyte ratios have been suggested as either markers of influenza susceptibility or disease severity in children [10,11,12]. In particular, the neutrophil/lymphocyte ratio is considered a flag of immune system homeostasis and has been suggested as part of the decision-making process concerning the admission/recovery rate of elderly patients with influenza [13,14].

Additionally, most studies on influenza seasonal outbreaks include only epidemiological data, with scarce information on clinical, radiological, and laboratory findings. The aim of this study was to describe the main characteristics of patients admitted to Cannes hospital for influenza during the 2024–2025 outbreak and to investigate risk factors for severe forms.

## 2. Materials and Methods

### 2.1. Study Design and Participants

We conducted an observational and retrospective study on patients admitted for influenza to either the Infectious Diseases Department or the Intensive Care Unit (ICU) in Cannes General Hospital from October 2024 to May 2025. Data from each patient were extracted from the hospital electronic database. The study was submitted to the internal register of the Clinical Research Department of Nice University Hospital; all patients received written information about the study and gave their consent to participate.

For each patient included, the following parameters were collected from their medical records and included in the analysis: demographics, underlying comorbidities, vaccination status, body mass index (BMI), symptom duration, clinical signs prior to admission, upon admission, and during hospitalization, laboratory and radiological findings during hospital stay, management, and clinical outcome.

Severe forms were defined by either death during the hospital stay or transfer to the ICU, while subjects admitted to the Infectious Diseases Department with a favorable outcome were defined as uncomplicated cases of influenza. The cause of death was also investigated.

According to the clinical outcome, two groups were identified: those with severe and those with uncomplicated influenza. The main characteristics of both groups were compared in order to measure any significant difference.

### 2.2. Laboratory Markers

The following measurements were collected and included in the statistical analysis: C-reactive protein, total white cell, neutrophil, hemoglobin, and platelet counts, and liver and renal function. Moreover, the neutrophil/lymphocyte (N/L) and platelet/lymphocyte (P/L) ratios upon admission were also calculated.

Diagnosis of influenza was performed by either a nasal or throat swab using NeuMoDx™ Flu A-B/RSV/SARS-CoV-2 (Qiagen, Courtaboeuf, France) or Xpert^®^ Xpress CoV-2/Flu/RSV (Cepheid, Maurens-Scopont, France) plus real-time RT-PCR assays.

### 2.3. Radiological Findings

Chest imaging results, either by radiography or computerized axial tomography, were collected for each patient and analyzed. According to the imaging results, patients were categorized as with or without pneumonia. In addition, rates of lung involvement above 50% of their surface were also measured.

### 2.4. Statistical Analysis

The main demographic, laboratory, and clinical characteristics and the outcome were described for the entire population. Categorical variables were illustrated as frequency rates and percentages, while continuous variables were detailed with medians and interquartile range (IQR).

Patients with severe and uncomplicated forms were compared using χ2-tests or Fisher’s exact test and the Wilcoxon–Mann–Whitney test. Variables with a *p*-value ≤ 0.10 in the univariate analysis were selected for the multivariate model.

Statistical analyses were performed using Statview^®^ software version 5.

## 3. Results

### 3.1. Patient Characteristics

From October 2024 to May 2025, we identified 59 subjects admitted for influenza to the Internal Medicine and Infectious Disease Department or to the ICU in Cannes hospital, Cannes, France (mean age 72 years, 56% male, 27% vaccinated, 93% with influenza type A, 66% with two or more comorbid conditions, 31% admitted to the ICU, and 14% deaths, as shown in Table 1). Only one of the patients received oseltamivir prior to admission, while 49 (83%) subjects received oseltamivir during their hospital stay. Among the fifty-five subjects with type A influenza, subtypes were available for nine patients, and all were admitted to the ICU, with six subjects harboring the H1N1 clade and three the H3N2 clade.

### 3.2. Risk Factors for Severe Influenza

Severe forms of influenza accounted for 22 subjects (37%), while 37 individuals had uncomplicated forms (63%). Among the eight deaths deplored during hospitalization, seven were certainly attributable to complications of influenza.

Comparison between subjects with severe and uncomplicated influenza showed that those with severe forms had higher N/L ratios upon admission, together with higher rates of acute hepatitis and more frequent pneumonia in chest imaging (Table 2). In particular, N/L means and medians among subjects with severe and uncomplicated forms allowed for the definition of different cut-offs for the multivariate analysis, with values below and above 15 showing the best performance for discriminating the two groups (Table 2 and Supplementary Table S1). Moreover, severe forms received oseltamivir more frequently during hospitalization (Table 2).

Although vaccination rates were higher among subjects with uncomplicated forms, the difference was not statistically significant (Table 2). Furthermore, no difference was found between groups in the case of pneumonia with lung involvement > 50% of their surface.

Multivariate analysis showed that an N/L ratio > 15 was an independent risk factor for severe influenza (OR_adj_ 7.98, 95% CI: 1.47–64.05, *p* = 0.024), as shown in Table 3. Although the N/L > 15 remained a significant predictive factor for severity, we did not include treatment with oseltamivir in the model, as we had extreme CI-95 values, probably because almost all subjects with severe forms received this compound.

The N/L > 15 yielded 40.9% sensitivity, 94.6% specificity, 81.8% positive predictive value, and 72.3% negative predictive value for predicting a severe form.

Treatment with oseltamivir did not influence the results of the multivariate analysis (Supplementary Table S1).

## 4. Discussion

We describe the main characteristics of patients admitted to the hospital for influenza during the 2024–2025 seasonal outbreak in France. To our knowledge, this is the first study to present the main clinical, radiological, and laboratory findings during the last influenza outbreak in Europe.

We found that an N/L ratio > 15 was an independent predictive marker of severe influenza, suggesting its usefulness for easily identifying subjects at higher risk for complicated forms. Indeed, such a marker, measured at admission, was associated both with severe forms upon arrival and with initially uncomplicated cases worsening during the hospital stay. Although sensitivity was low, this test showed good accuracy in terms of specificity, VPP, and VPN, suggesting its role in ruling out a complicated form in case of a negative result.

The N/L ratio is a simple biomarker of systemic inflammation, which combines both innate and adaptive immunity [15]. Indeed, neutrophils reflect mostly innate immune functions, with a continuous crosstalk with adaptive immunity, mediated by lymphocytes. In fact, neutrophils are the first leucocytes to be recruited at the infection site to enhance local innate responses. They are also able to produce large amounts of cytokines, which may influence many aspects of the immune response and can also serve as antigen-presenting cells for CD8+ lymphocytes [15,16].

When stimulated by various antigens, including bacteria, viruses, and cytokines, neutrophils could release chromatin-based structures, called neutrophil extracellular traps (NETs), which, at high levels, have been associated with lung damage during influenza infection [17,18].

The influenza virus is known for causing lymphopenia in CD4+, CD8+, and NK+ cell subsets via various mechanisms, including cellular apoptosis, involvement of cytokines, chemokines, and growth factors, inhibition of lymphopoiesis, lymphocyte sequestration in lungs, and release of co-inhibitory molecules [19]. Considering that lymphocytes are critical for viral clearance, lymphopenia may thus affect the host’s adaptive immune response and may impact the clinical course of influenza.

The fact that such a ratio was associated with severe forms, independent of lung radiological involvement, argues, in our view, in favor of a direct effect of the virus on these parameters rather than simply reflecting the secondary bacterial pneumonia associated with influenza.

Interestingly, although information on the influenza vaccine status was not available for the entire population, vaccination rates were lower than those recommended by national and international guidelines [20,21]. However, we did not find any protection by vaccination for severe forms. Such results require very prudent conclusions, considering the small number of subjects and the study design, but they suggest that the unavailability of the high-dose influenza vaccine in France during the 2024–2025 vaccine campaign could partially explain the poor protection conferred by immunization. Indeed, national guidelines have been recently modified, and a trivalent high-dose influenza vaccine will be available again for the 2025–2026 campaign [22].

Additionally, although the study was not designed to measure antiviral efficacy, treatment with oseltamivir was not associated with a better outcome, despite the short duration of symptoms in both groups. This result could be related to moderate antiviral efficacy on severe influenza, as suggested by Gao et al., who recently showed little or no difference in time to alleviation of symptoms with either oseltamivir or peramivir when compared to placebo [23]. Alternatively, as suggested mechanisms explaining clinical severity include cytokine storm, vasoconstriction, and lung damage [24], anti-inflammatory and antiviral compounds could both be necessary. Although non-steroidal anti-inflammatory drugs have been associated with worse outcomes [25], other interesting anti-inflammatory molecules, such as Taurolidine and Janus kinase inhibitors, showed promising results [24,26]. Therefore, we suggest that the N/L ratio could feature prominently in the future not only as a useful marker for decision-making in patients hospitalized for influenza but also as a valuable factor in selecting candidates for novel anti-inflammatory strategies.

Limitations of the study include, together with the small number of subjects, its retrospective character and the lack of distinction between the H1N1 and the H3N2 clades, which would have been helpful to investigate potential differences in the predictive value of the N/L ratio differs according to the virus subtype.

## 5. Conclusions

In conclusion, we found that the N/L ratio > 15 is a predictive risk factor for disease severity in patients admitted to hospital for influenza during the seasonal 2024–2025 outbreak, warranting larger prospective studies.

## Figures and Tables

**Table 1:** Patient characteristics.

	N (%) or Median [IQR]
**Number of patients**	59
**Male gender**	33 (55.9%)
**Age (years)**	73.0 [62.0; 82.5]
**Body mass index**	23.1 [20.2; 25.8]
**Days of symptoms prior to diagnosis**	3.0 [1.0; 5.0]
**Comorbid conditions**	
Less than 2	20 (33.9%)
More than 2	39 (66.1%)
**Influenza type**	
A	55 (93%)
B	4 (7%)
**Previous flu vaccination** *	13 (26.5%)
**Neutrophil/lymphocyte ratio**	7.1 [3.7; 12.6]
**Neutrophil/lymphocyte ratio (mean [SD])**	10.3 [12.3]
**Platelet/lymphocyte ratio**	228.4 [118.3; 344.3]
**Acute renal failure**	22 (37.2%)
**Acute hepatitis**	23 (38.9%)
**Pneumonia in chest imaging**	30 (51.7%)
**Admissions to Intensive Care Unit**	18 (30.5%)
**Deaths**	8 (13.6%)

* Information available only for 42 subjects.

**Table 2:** Comparison between patients with severe and uncomplicated influenza.

	Severe	Uncomplicated	*p*-Value
	N (%) or Median [IQR]	N (%) or Median [IQR]	
**Number of patients**	22	37	
**Male gender**	13 (59.1%)	20 (54.1%)	0.706
**Age (years)**	70.5 [62.3; 78.8]	76.0 [62.0; 84.0]	0.293
**ASAT**	63.5 [31.3; 158.3]	40.0 [26.5; 65.3]	0.034
**ALAT**	35.0 [22.5; 60.5]	19.5 [14.0; 31.0]	0.013
**More than 2 comorbid conditions**	6 (27.2%)	14 (37.8%)	0.407
Vaccination for influenza *	3 (17.6%)	10 (40.0%)	0.124
**Acute renal failure**	11 (50.0%)	11 (29.7%)	0.119
**Acute hepatitis**	12 (54.5%)	11 (30.5%)	0.070
**Baseline C-reactive protein value (mg/L)**	87.0 [67.0; 185.0]	86.0 [38.0; 173.3]	0.432
**Baseline N/L ≥ 7**	13 (59.1%)	17 (45.9%)	0.329
**Baseline N/L ≥ 10**	11 (50.0%)	8 (21.6%)	0.024
**Baseline N/L > 15**	9 (40.9%)	2 (5.4%)	0.001
**Baseline P/L > 300**	9 (40.9%)	11 (29.7%)	0.380
**Pneumonia in chest imaging**	14 (63.6%)	14 (37.8%)	0.055
**Treatment with oseltamivir**	21 (95.4%)	28 (75.7%)	0.074

* Available only for 42 subjects. N: neutrophils. L: lymphocytes. P: platelets.

**Table 3:** Independent risk factors for severe influenza.

		Severe vs. Uncomplicated Influenza		
		Adjusted OR	CI-95%	*p*-Value
ASAT		1.00	[0.98; 1.02]	0.880
ALAT		1.02	[0.98; 1.06]	0.373
N/L ratio > 15		7.98	[1.47; 64.05]	0.024
Pneumonia in chest imaging		2.33	[0.66; 8.55]	0.188

ASAT: aspartate aminotransferase; ALAT: alanine aminotransferase; N/L: neutrophils/lymphocytes; P/L: platelets/lymphocytes.

## Data Availability

The original contributions presented in this study are included in this article; further inquiries can be made to the corresponding author.

## Supplementary Materials

The following supporting information can be downloaded at https://www.mdpi.com/article/10.3390/idr17050127/s1: Complete univariate and multivariate analysis, full dataset, and histogram of values distribution.
