# Supplementary material for: The Neutrophil/Lymphocyte Ratio Was Identified as a Marker of Severe Influenza During the 2024–2025 Outbreak in France
Source: Infect Dis Rep. 2025 Oct 10;17(5):127. doi: 10.3390/idr17050127 (PMC12562332; doi:10.3390/idr17050127)
Supplement: Supplementary file 1 [file idr-17-00127-s001.zip › Table S1 Complete_univariate and multivariate supplementary final.pdf]

		Severe - n=22				Uncomplicated - n=37				
	n	Mean	[SD]	Median	[Q1 ; Q3]	Mean	[SD]	Median	[Q1 ; Q3]	p-value
Body Mass Index	54	24.1	[5.6]	24.2	[21.6 ; 26.2]	23.1	[4.2]	22.9	[20.1 ; 25.1]	0.546
Days of symptoms prior to diagnosis	52	3.2	[2.3]	3.0	[1.0 ; 5.0]	3.7	[2.6]	3.0	[2.0 ; 5.0]	0.556
Fibrinogen	28	5.8	[2.2]	5.3	[4.5 ; 7.2]	4.8	[1.4]	4.8	[3.8 ; 5.4]	0.161
Baseline C-reactive protein value (mg/L)	57	128.7	[104.2]	87.0	[67.0 ; 185.0]	108.7	[90.6]	86.0	[38.0 ; 173.3]	0.432
Leucocytes	59	10.6	[7.5]	8.1	[5.5 ; 12.6]	8.4	[4.5]	7.3	[5.5 ; 10.5]	0.549
Neutrophils	59	8.8	[6.7]	7.2	[4.2 ; 10.3]	6.3	[3.5]	5.4	[3.9 ; 7.8]	0.338
Lymphocytes	59	0.8	[0.6]	0.7	[0.4 ; 1.1]	2.0	[2.8]	1.0	[0.6 ; 1.7]	0.047
Platelets	59	193.0	[76.8]	179.5	[137.0 ; 267.5]	188.5	[84.3]	187.0	[124.0 ; 241.0]	0.969
ASAT	58	109.3	[131.1]	63.5	[31.3 ; 158.3]	49.2	[35.6]	40.0	[26.5 ; 65.3]	0.034
ALAT	58	51.5	[49.3]	35.0	[22.5 ; 60.5]	26.2	[18.6]	19.5	[14.0 ; 31.0]	0.012
Fi02 (%)	27	0.5	[0.3]	0.5	[0.3 ; 0.6]	21.0	[0.0]	21.0	[21.0 ; 21.0]	0.000
Age (years)	59	69.5	[14.1]	70.5	[62.3 ; 78.8]	72.9	[15.2]	76.0	[62.0 ; 84.0]	0.293
Fibrosis 4 index (FIB-4)	58	6.8	[6.7]	5.0	[2.1 ; 10.1]	12.0	[44.3]	4.0	[2.4 ; 6.5]	0.540
Baseline N/L ratio	59	16.3	[17.8]	10.2	[5.0 ; 18.5]	6.7	[5.0]	6.2	[2.5 ; 9.4]	0.011
Baseline P/L ratio	59	329.9	[213.9]	253.0	[192.6 ; 430.6]	215.2	[150.6]	220.0	[93.3 ; 324.0]	0.063
	n valide			n	(%)			n	(%)	p-value
**Gender**	59									0.706
Female				9	(40.9)			17	(45.9)	
Male				13	(59.1)			20	(54.1)	
**Influenza type**	59									0.141
A				19	(86.4)			36	(97.3)	
B				3	(13.6)			1	(2.7)	
**Co-infection**	43									0.024
No				3	(30.0)			24	(72.7)	
Yes				7	(70.0)			9	(27.3)	
**Acute hepatitis**	58									0.070
No				10	(45.5)			25	(69.4)	
Yes				12	(54.5)			11	(30.6)	

**Pneumonia at chest imaging**	59			0.055
No		8 (36.4)	23 (62.2)	
Yes		14 (63.6)	14 (37.8)	
**Lung involvement > 50%**	56			0.024
No		16 (76.2)	34 (97.1)	
Yes		5 (23.8)	1 (2.9)	
**Overweight**	56			0.426
No		17 (81.0)	25 (71.4)	
Yes		4 (19.0)	10 (28.6)	
**Obesity**	56			0.626
No		19 (90.5)	33 (94.3)	
Yes		2 (9.5)	2 (5.7)	
**Vaccination for influenza**	42			0.124
No		14 (82.4)	15 (60.0)	
Yes		3 (17.6)	10 (40.0)	
**Age**	59			0.451
< 65		8 (36.4)	10 (27.0)	
> 65		14 (63.6)	27 (73.0)	
**Comorbid conditions**	59			0.407
2 or more		6 (27.3)	14 (37.8)	
0 or 1		16 (72.7)	23 (62.2)	
**Baseline N/L ratio**	59			0.329
< 7		9 (40.9)	20 (54.1)	
≥ 7		13 (59.1)	17 (45.9)	
**Baseline N/L ratio**	59			0.024
< 10		11 (50.0)	29 (78.4)	
≥ 10		11 (50.0)	8 (21.6)	
**Baseline N/L ratio**	59			0.001
< 15		13 (59.1)	35 (94.6)	
> 15		9 (40.9)	2 (5.4)	

**Baseline P/L ratio**	59			0.380
< 300		13 (59.1)	26 (70.3)	
> 300		9 (40.9)	11 (29.7)	
**Acute renal failure**	59			0.119
No		11 (50.0)	26 (70.3)	
Yes		11 (50.0)	11 (29.7)	
**Treatment with oseltamivir**	59			0.074
No		1 (4.5)	9 (24.3)	
Yes		21 (95.5)	28 (75.7)	

Complete univariate analysis

	Severe vs uncomplicated influenza		
	Adjusted OR	CI-95%	p-value
ASAT	1.00	[0.98 ; 1.03]	0.690
ALAT	1.02	[0.98 ; 1.08]	0.445
N/L ratio > 15	12.58	[1.77 ; 257.36]	0.028
Pneumonia at chest imaging	1.53	[0.38 ; 6.15]	0.540
Treatment with oseltamivir	98.57	[2.30 ; 6.61*10^5]	0.132

Multivariate analysis including treatment with oseltamivir
