# Supplementary figures and images for: The Neutrophil/Lymphocyte Ratio Was Identified as a Marker of Severe Influenza During the 2024–2025 Outbreak in France
Source: Infect Dis Rep. 2025 Oct 10;17(5):127. doi: 10.3390/idr17050127 (PMC12562332; doi:10.3390/idr17050127)

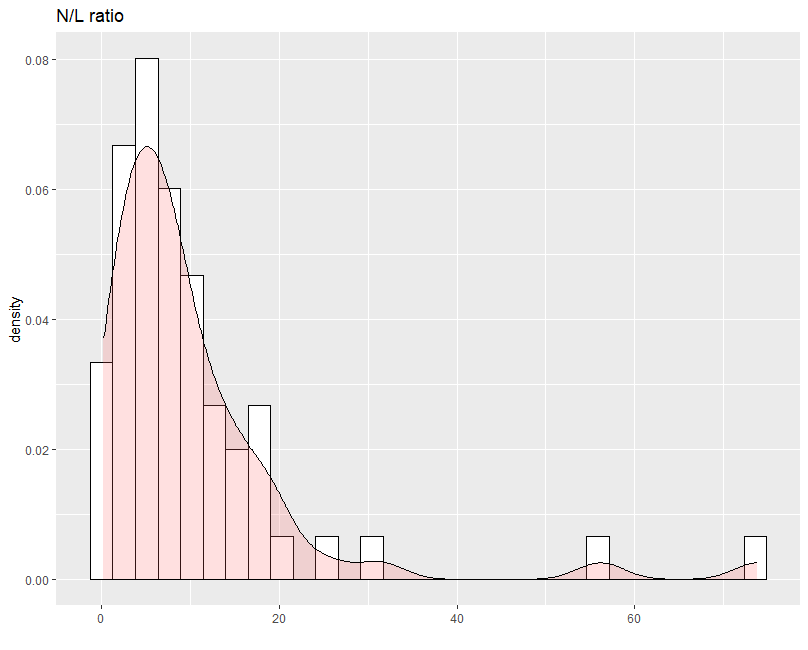

Supplement: Supplementary file 1 [file idr-17-00127-s001.zip › Fig S1 histo_nl_ratio.png]
